# Antibodies against Immature Virions Are Not a Discriminating Factor for Dengue Disease Severity

**DOI:** 10.1371/journal.pntd.0003564

**Published:** 2015-03-11

**Authors:** Izabela A. Rodenhuis-Zybert, Júlia M. da Silva Voorham, Silvia Torres, Denise van de Pol, Jolanda M. Smit

**Affiliations:** Department of Medical Microbiology, University of Groningen and University Medical Center Groningen, Groningen, The Netherlands; University of North Carolina School of Medicine, Chapel Hill, NC, UNITED STATES

## Abstract

Humoral immunity plays an important role in controlling dengue virus (DENV) infection. Antibodies (Abs) developed during primary infection protect against subsequent infection with the same dengue serotype, but can enhance disease following secondary infection with a heterologous serotype. A DENV virion has two surface proteins, envelope protein E and (pre)-membrane protein (pr)M, and inefficient cleavage of the prM protein during maturation of progeny virions leads to the secretion of immature and partially immature particles. Interestingly, we and others found that historically regarded non-infectious prM-containing DENV particles can become highly infectious in the presence of E- and prM-Abs. Accordingly, we hypothesized that these virions contribute to the exacerbation of disease during secondary infection. Here, we tested this hypothesis and investigated the ability of acute sera of 30 DENV2-infected patients with different grades of disease severity, to bind, neutralize and/or enhance immature DENV2. We found that a significant fraction of serum Abs bind to the prM protein and to immature virions, but we observed no significant difference between the disease severity groups. Furthermore, functional analysis of the Abs did not underscore any specific correlation between the neutralizing/enhancing activity towards immature DENV2 and the development of more severe disease. Based on our analysis of acute sera, we conclude that Abs binding to immature virions are not a discriminating factor in dengue pathogenesis.

## Author Summary

The four serotypes of the mosquito-borne dengue virus (DENV) cause an estimated 390 million human infections per annum. Symptomatic infection can manifest itself as a self-limiting febrile illness, dengue fever (DF), or as more severe and potentially life-threatening dengue hemorrhagic fever (DHF), and dengue shock syndrome (DSS). Severe disease development is usually associated with the presence of pre-existing Abs that enhance DENV infection rather than neutralize it. Antibody-dependent enhancement of infection is believed to contribute to high viral loads that prelude the development of severe disease. Indeed, Abs binding to the DENV surface glycoproteins E and prM are known to enhance infection. Here, we studied the role of prM Abs and prM-containing immature virions in the pathogenesis of severe disease. We analyzed the ability of acute sera of DF, DHF and DSS patients to bind, neutralize and/or enhance immature DENV infection. We found that a significant fraction of Abs bind to prM protein of DENV2; however, there was no difference between the disease severity groups. Moreover, we did not observed any specific correlation between the neutralizing/enhancing activity towards immature DENV2 and disease presentation. Based on these data we inferred that prM Abs and immature virions are not a discriminating factor in dengue pathogenesis. These findings are important for the understanding of dengue pathogenesis and the development of new vaccines.

## Introduction

The four serotypes of dengue virus (DENV1–4) currently represent the most prevalent mosquito-borne viral disease of humans. It has been recently estimated that 390 million infections occur each year, mainly in the (sub)tropical regions of the world [1–3]. Symptomatic infection with any of the four DENV serotypes manifests as dengue fever (DF), a self-limiting but debilitating febrile illness or the more severe and potentially life-threatening dengue hemorrhagic fever (DHF) and dengue shock syndrome (DSS) [4]. Pathogenesis of DENV infection is multifaceted [5], but sequential infection with a heterologous DENV serotype as well as primary infection of infants born to dengue-immune mothers are known risk factors for severe disease. Accordingly, it has been hypothesized that Abs generated during primary infection or passively acquired DENV-specific maternal IgG can exacerbate the symptoms of infection *via* antibody-dependent enhancement (ADE) of disease. In ADE, cross-reactive, non-neutralizing Abs facilitate successful entry of virus-immune complexes into Fcγ-receptor-bearing cells [6–8] leading to an increased viral load early in infection [9, 10] and ultimately to the exacerbation of disease [11]. *In vitro*, ADE of DENV infection can be observed in many Fc-receptor-expressing cell lines, including K562, U937, P388D1 and in primary human cells such as monocytes, macrophages and mature dendritic cells [6, 12, 13].

The viral surface of mature DENV is covered with 180 copies of 2 transmembrane glycoproteins: the membrane (M) and envelope (E) protein. The ectodomain of the E protein has three structurally distinct domains (DI, DII, DIII) and mediates cell entry of the virion into target cells [14–17]. Within infected cells progeny virions are assembled in an immature form. These immature virions are structurally distinct from mature particles and contain 180 heterodimers of the E and precursor (pr)M protein anchored in the viral membrane. In this conformation, prM stabilizes the E protein thereby inhibiting its fusogenic activity [18]. Immature DENV virions mature during transit through the Golgi and trans-Golgi network, where the cellular protease furin cleaves prM to M and a “pr” peptide. The “pr” peptide is released upon secretion of the particle into the pH neutral extracellular milieu [18]. Importantly, the maturation process of DENV appears to be inefficient as infected cells release significant numbers of immature and partially mature virions [19, 20].

The E protein is the principal target of neutralizing and enhancing Abs developed in response to DENV infection [21–23]. A large proportion of the human Abs is directed against the fusion loop in EDII [24] and *in vitro* analysis has revealed that many of these Abs possess weakly neutralizing and highly cross-reactive properties [21, 22, 25]. Only a small fraction of the human anti-DENV antibody response consists of highly neutralizing Abs, which appear to be binding to EDII [26–28] or to a complex epitope spanning EDI–EDII [22, 29]. Furthermore, and in line with the presence of immature virions within standard (std) DENV preparations *in vitro* [19, 20], prM-Abs are generated during primary and secondary DENV infections [23, 29]. Notably, several studies reported significantly higher levels of prM-Abs in secondary infections when compared to primary infections [21, 30]. Antibodies directed against prM generally possess non- or weakly neutralizing properties and are highly cross-reactive between DENV serotypes [22, 23, 29].

It has been shown that prM and E Abs render the historically regarded non-infectious fully immature DENV particles highly infectious in Fc-receptor bearing cells [23, 31–34]. Antibody-opsonized immature DENV binds to Fc-receptors and this interaction facilitates cell entry of the immune complex *via* an as yet unknown pathway. The acidic environment of endosomes/phagosomes subsequently triggers conformational changes in the virion allowing furin to cleave the prM protein [31]. Furthermore, the low pH conditions in the late endocytic/phagocytic pathway (to approximately 5.0) is thought to prompt the release of “pr” peptide thereby rescuing the fusion of the virions with the endosomal membrane and release of the infectious RNA into the cytoplasm [35, 36]. Accordingly, in presence of Abs, immature particles expand the pool of circulating infectious virus thereby possibly contributing to DENV pathogenesis [23, 31–33].

In this study we investigated the potential role of immature DENV in disease pathogenesis. First, we examined the antigen-specificity of Abs in sera of patients with acute DENV2 infection and compared the prM- to E- Abs ratio in relation to disease severity (DF, DHF, DSS). Next, we tested the capacity of the sera to neutralize and/or enhance the current infecting DENV serotype. We show that prM-Abs were present in all immune sera tested. Moreover, immune sera from all severity groups were able to bind to and enhance infectivity of immature DENV2 particles. Notably, antibody-mediated enhancement of std DENV2 infection by the sera partially relied on enzymatic activity of furin. However, in this study, we found no significant difference between the 3 disease groups, suggesting that Abs recognizing immature DENV virions cannot be used as a predisposing factor for severe disease development.

## Materials and Methods

### Patients

Immune sera were obtained from the ongoing Pediatric Hospital-based Dengue Study in Nicaragua [37]. DF, DHF and DSS were defined according to the 1997 WHO classification scheme [4]. All patients were confirmed as dengue-positive by RT-PCR, virus isolation, IgM seroconversion and/or a ≥4-fold rise in antibody titer as measured by Inhibition ELISA [37–40]; DENV2 infection was confirmed by RT-PCR [41, 42]. All patients had experienced a secondary DENV infection with an antibody titer of ≥10 (acute-phase) or ≥2,560 (convalescent-phase) as determined by Inhibition ELISA [37, 40, 43, 44]. The sera used in this study were collected 2 to 4 days after the onset of fever. In total, 30 patients: 10 with DF, 10 with DHF and 10 with DSS were analyzed.

### Ethics statement

The study was approved by the Institutional Review Boards of the University of California, Berkeley, and of the Nicaraguan Ministry of Health. Parents or legal guardians of all subjects provided written informed consent, and subjects 6 years of age and older provided assent.

### Cells

*Aedes albopictus* C6/36 cells were maintained in minimal essential medium (Life Technologies) supplemented with 10% fetal bovine serum (FBS), 25 mM HEPES, 7.5% sodium bicarbonate, penicillin (100 U/ml), streptomycin (100 μg/ml), 200 mM glutamine and 100 μM nonessential amino acids at 30°C and 5% CO_2_. Baby Hamster Kidney clone 15 cells (BHK21–15) cells were cultured in DMEM (Life Technologies) containing 10% FBS, penicillin (100 U/ml), streptomycin (100 μg/ml), 10 mM HEPES, and 200 mM glutamine. Human adenocarcinoma LoVo cells were cultured in Ham’s medium (Invitrogen) supplemented with 20% FBS at 37°C and 5% CO_2_. Mouse macrophage P388D1 cells were maintained in DMEM supplemented with 10% FBS, penicillin (100 U/ml), and streptomycin (100 μg/ml), sodium bicarbonate (Invitrogen, 7,5% solution) and 1.0 mM sodium pyruvate (GIBCO) at 37°C and 5% CO_2_.

### Virus growth

DENV2 strain 16681 was propagated in C6/36 cells, as described previously and is referred to as standard (std) DENV2. [19, 45]. Immature (prM) DENV2 was produced in LoVo cells and characterised as described previously [19]. Briefly, LoVo cells were infected at a multiplicity of infection (MOI) of 5, and 1.5 hours post-infection (hpi) the virus inoculum was removed, cells were washed three times with PBS, and fresh medium was added. At 72 hpi, the medium containing the virus particles was harvested, cleared of cellular debris by low-speed centrifugation, aliquoted, and stored at -80°C. To assure the immature status of the virus preparation the specific infectivity of the LoVo-derived DENV2 was determined by measuring the number of infectious units by plaque assay on BHK21–15 cells and the number of genome-containing particles (GCPs) by quantitative PCR (qPCR) analysis, as described previously [45]. As expected, the specific infectivity of prM DENV2 was at least 10000–fold lower than that of std DENV2 preparation.

### Antibody specificity assays

The binding properties of dengue-immune sera to immature virus particles was assessed by Western Blot (WB) and indirect ELISA.

For Western blot analysis, 1.0x10^9^ GCPs of purified immature DENV2 was loaded on 12.5% SDS polyacryramide gels under non-reducing conditions. The blot was incubated with 3 serial dilutions (fold dilution was dependent on the IgG concentration in the sera) of 30 individual sera or with pooled sera from 10 DF, 10 DHF or 10 DSS patients. A human monoclonal prM antibody (hmAb) 75.9 (a kind gift from A. Lanzavecchia, Institute for Research in Biomedicine, Bellinzona, Switzerland) was used as a positive control. The antibody response against the E and prM protein was analyzed with ImageQuant TL. The percentage of prM protein is calculated by relating the intensity of the prM band to the total intensity of prM and E (examples of WB and the quantifications are added as S1 Fig). Values obtained from three independent experiments were compared by Mann-Whitney test and ANOVA.

The binding properties of dengue-immune sera to immature virus particles was assessed by indirect ELISA. Briefly, microtiter ELISA plates (Greiner bio-one) were coated overnight with 1x10^8^ GCPs of purified virus per well in 100 μl coating buffer. After blocking with 2% milk in coating buffer for 120 min, 100 μl of two-fold serial dilutions in PBS of controls (2 dengue-negative sera, and PBS alone), and individual or pooled dengue- immune sera from patients with DF, DHF and DSS were applied to the wells and incubated for 1.5 h, in triplicate. Subsequently, after extensive washing, 100 μl of horseradish peroxidase-conjugated mouse anti-human IgG antibody (1:5000, Southern Biotech) or was applied for 1 h. To measure serum IgM, a donkey anti-human IgM, Fc-fragment-specific mAb (1:4000, ABD Serotec; Biorad) conjugated to horseradish peroxidase was used as secondary Ab. All incubations were performed at 37°C. The substrate o-phenylene-diamine (OPD) (Eastman Kodak Company) was added and absorbance was read at 492 nm (A492) with an ELISA reader (Bio-tek Instruments, Inc.). The highest positive (cut off = mean control sample OD + 3xSTDEV of PBS control) in the assay was defined as the endpoint titer. Endpoint titers for dengue-negative sera were lower than 1:1600 for both std- and prM- DENV2. Endpoint titers from at least 4 experiments were used to calculate geometrical mean titer (GMT) of dengue positive sera.

### Avidity assay

To determine serum IgG avidity ELISA was performed as described above, with the following modifications. First, the pooled sera were added at a dilution with an expected absorbance of 1.0 ± 0.2 to reach the linear part of the titration curve. Second, after the sera were incubated for 1,5 h, 8M urea was added for 10 minutes instead of PBS before adding the secondary Ab [46]. This concentration of urea was chosen because our preliminary experiments showed that urea concentrations of >8 M always markedly reduced the OD value even if the urea was added, incubated, and washed before the reaction with the sera. This suggested at the higher concentrations urea can cause a falsely low reading by removing the coated DENV2 virions. The avidity index was defined as the ratio of the OD with urea to the OD without urea.

### Infectivity assays

Virus or virus-immune complexes were added to a monolayer of P388D1 cells (2 × 10^5^) in 24-well plates (Costar) at a multiplicity of genome-containing particles (MOG) per cell of 500. At 1.5 hpi, fresh medium was added to the cells. At 43 hpi (time points between 24–43hpi represent single round of infection as determined by quantifying dengue-positive cells using flow cytometry), the medium was harvested and virus production was analyzed by plaque assay on BHK21–15 cells, as described previously [47]. The limit of detection in the plaque assay is 20 PFU/ml. In experiments where furin activity was transiently blocked, cells were treated with 25 μM of furin-specific inhibitor, decanoyl-L-arginyl-L-valyl-L-lysyl-L-arginyl-chloromethylketone (decRRVKR-CMK) (Calbiochem) prior to and during virus infection according to our previously published protocol [31]. Infection with std DENV2 with and without FI was used as a control to assess the effect of the FI on the maturation of progeny virions.

## Results

### A significant fraction of human Abs is directed against the prM protein

**Fig 1:**
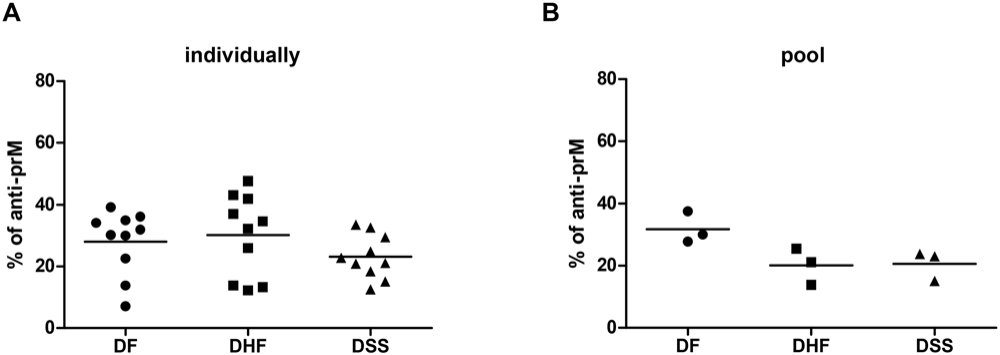
Specificity of human antibody response against DENV2. Western Blot analysis of sera IgG binding to purified immature (prM) DENV2 proteins. Relative percentage of prM is shown for (A) individual serum samples within the 3 severity groups and (B) pooled sera from 10 DF, 10 DHF or 10 DSS patients. Data are representative of at least 3 independent experiments. Mann-Whitney test was used to compare DF with DHF or DSS, no significant difference was observed.

First, we investigated if the prM-specific antibody response is more abundant in patients with severe disease. To this end, we loaded immature DENV2 virions onto a SDS-PAGE gel and performed WB analysis using acute serum from patients with secondary DENV2 infections presenting different grades of severity. The specific antibody response and the ratio of prM and E Abs in individual and pooled serum samples was quantified by means of ImageQuant TL software, as described in Materials and Methods. We first verified that there were no differences in the individual serum IgG levels between the 3 studied groups (Kruskal-Wallis statistic; S2 Fig). We focused on the measurement of immature (prM) DENV2- reactive IgG (Fig 1) as these are most prevalent during secondary infection. The IgM response is usually detectable very early in secondary infection but in our samples set (2–4 days post infection) the IgM signal was too low to demonstrate differences between the groups (S3 Fig) [48]. Irrespective of whether we analyzed samples individually (Fig 1A) or as a DF, DHF or DSS pool (Fig 1B), approximately 20–35% of the measured IgG response was directed to the prM protein. These data suggest that a robust prM antibody response present during a secondary infection may not be indicative of severe disease development.

### Dengue specific antibodies recognize immature virions

**Fig 2:**
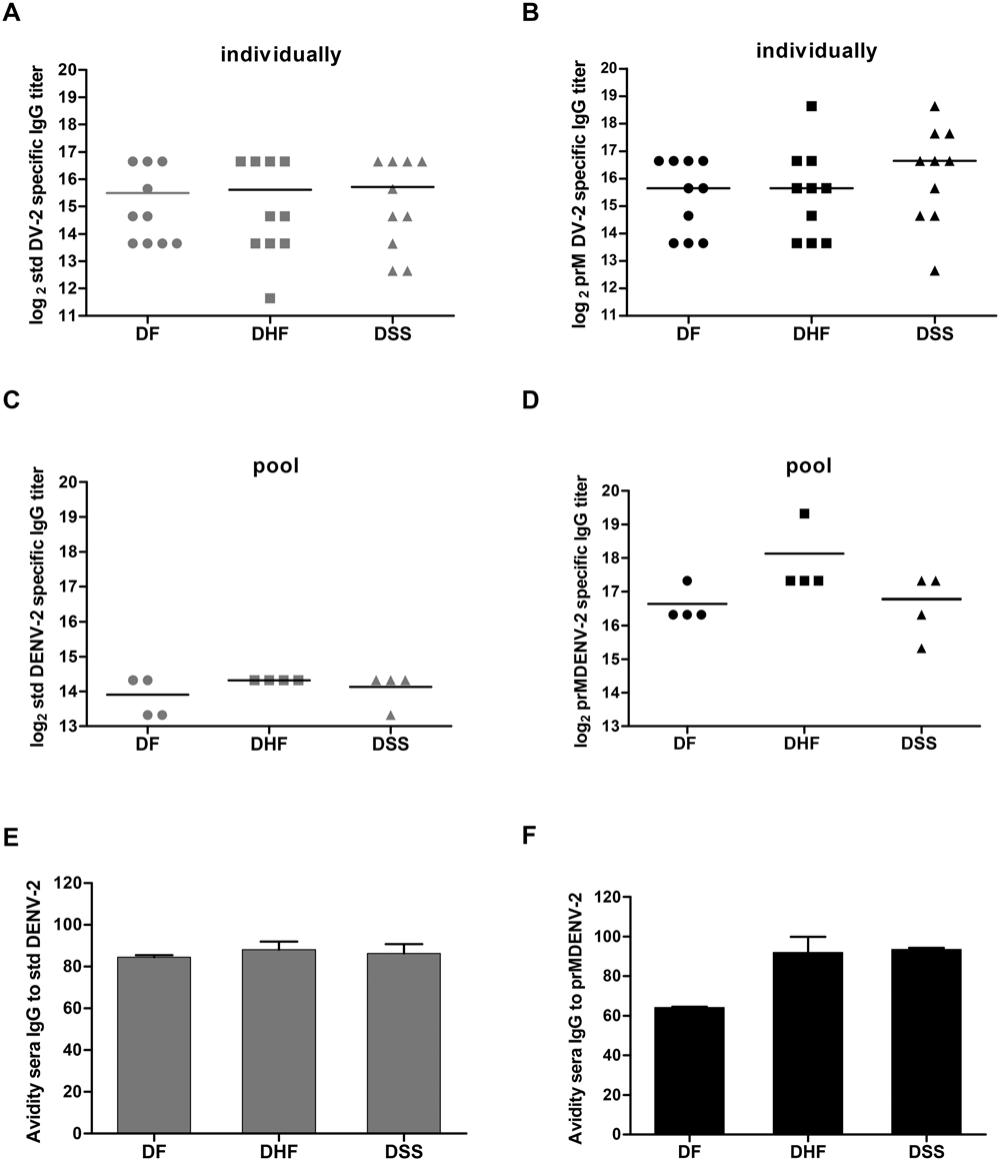
Capacity of immune sera to bind immature and standard DENV2. Binding of sera IgG to standard (std) DENV2 and immature (prM) DENV2 was analyzed by means of indirect ELISA. (A) and (B) represent the GMT of each individual serum for std and prM DEMV-2 respectively. (C) and (D) represent the GMT obtained with the pooled serum samples in four separate experiments. One way Anova and Mann Whitney was used to compare the groups, no significance was found. (E) and (F) depict the avidity of binding of the 3 sera pools to std DENV2 and prM DENV2, respectively. The data represent the mean and the standard error of the mean (SEM) of 2 experiments performed in triplicate.

As both prM and E Abs may be conformation sensitive [21, 49] the detection of certain IgG clones could be underestimated by means of WB technique [29]. To rule this out, we next analyzed the capacity of the human IgG to react with std DENV2 and immature (prM) preparations by means of indirect ELISA, as described in Material and Methods (Fig 2). Panels 2A and 2B depict representative reciprocal end-point titers of individual serum samples that bind to std and prM DENV2, respectively. Panels C and D represent the data obtained with pooled serum samples. Regardless of the analysis method used, immune sera from all three disease severity groups contained similar levels of Abs that bind to std DENV2 (Fig 2A,C) and immature prM DENV2 (Fig 2B,D). The GMT of IgG binding to immature DENV2 was found significantly higher (p = 0.0012, One way Anova, Kruskal-Wallis test) when assessing pooled serum samples. We do not fully understand why this difference is not as apparent when analyzing individual samples however it is likely that individual serum samples with high GMT of IgG binding to prM have confounded the pooled sera result. Importantly however, this phenomenon had no effect on the differences between the groups. Also, in line with the WB results, we did not detect a significant difference in the relative binding of prM and std DENV2 between the severity groups.

To characterize the potential difference in the strength of the Abs-Ag binding that could potentially result in different neutralization capacities, we also measured the avidity of DENV-specific sera pools to prM and std DENV2 using a modified ELISA with urea washes. Avidity was defined as the percentage (%) of IgG that remained bound after the urea washes. We found no difference in the avidity of the sera to neither std DENV2 (Fig 2E) nor to prM DENV2 (Fig 2F). Although DF sera had a lower avidity to prM DENV2 (average of approximately 65%) than that of DHF/DSS (average of approximately 90%), this difference was not statistically significant when assessed by One way Anova and Mann-Whitney test.

### Dengue-immune sera from all severity groups promote infectivity of immature DENV2

**Fig 3:**
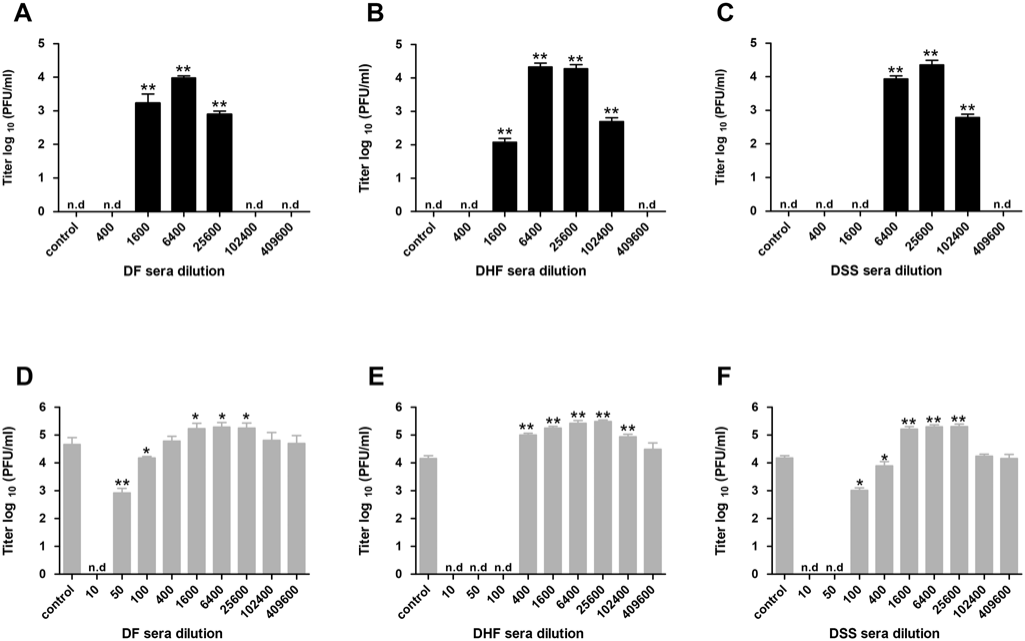
Immune sera neutralize and enhance the immature and std DENV2 infection. P388D1 cells were infected with (A-C) prM or (D-F) std DENV2 at MOG 500 in the presence or absence of serially diluted pooled-sera. At 43 hpi supernatant was harvested and virus production was analyzed by plaque assay on BHK21–15 cells. Panel (A) and (D) represent data for DF pool; panel (B) and (E), data for DHF pool; and panel (C) and (F) for DSS pool. Data are expressed as means of at least two independent experiments performed in triplicate. The error bars represent SEM; (n.d.) denotes ‘‘not detectable”. Mann-Whitney test were used to compare infection with and without each sera dilution; * = *P* < 0.05; ** = P < 0.01.

Having established that neither the quantity nor the avidity of immature-virus specific Abs could distinguish disease severity, we next evaluated the protective value of these responses. The infectivity of std and prM DENV2 preparations was measured in the absence and presence of serum in murine macrophage-like P388D1 cells, which express three distinct Fc-γ-receptors (FcγR), FcγRIII [CD16], FcγRII [CD32], and FcγRI [CD64]) [50, 51]. After 1 round of infection (see Material and Methods), we harvested the supernatant and analyzed the infectious virus production. Initially, we tested we capacity of all individual serum samples to neutralize and/or enhance DENV2 infection. However, as no significant differences were found (ADE of prM DENV2 is shown in S4 Fig), we decided to use pooled serum samples in further experiments (Fig 3). We observed that immune sera of all severity groups neutralized immature DENV2 particles at a dilution of 400x or lower. Enhancement of infectivity was observed at higher dilutions 1600x-102400x (Fig 3A–C). Notably, at highest power of enhancement, the infectious titer of prM DENV2-immune complexes reached a level comparable to that of std DENV2 in the absence of immune sera. These findings are consistent with our previous work, which demonstrated that immature DENV particles are infectious in the presence of polyclonal sera, anti-prM and anti-E Abs [23, 31–33]. Furthermore, and as expected, immune sera from all severity groups enhanced std DENV2 infection (Fig 3D,E,F), albeit with a lower power of enhancement when compared to that of immature particles. Interestingly, DHF sera were most efficient in neutralizing std DENV2 infectivity at high sera concentrations but also elicited ADE over the broadest range of sera dilutions (Fig 3E).

### Immature particles play a significant role in ADE of std DENV2 elicited by immune sera of all three disease severity groups

**Fig 4:**
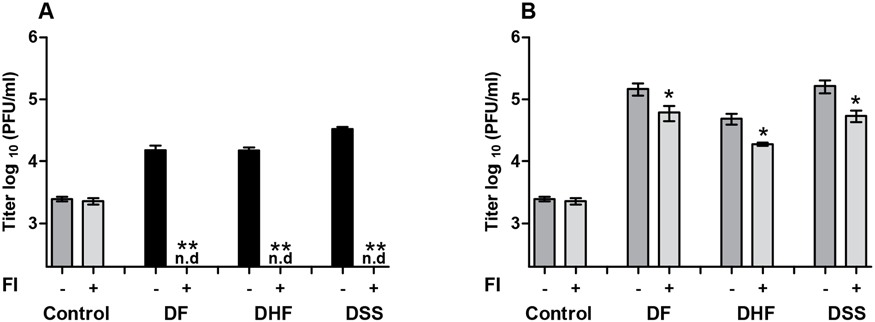
Immature particles play a significant role in ADE of std DENV2 elicited by all three severity groups of immune sera. Non-treated P388D1 cells and cells treated with furin inhibitor (FI) were infected with immature (A) and std (B) DENV2 at MOG 500 in the absence or presence of pooled immune sera at 6400x dilution. Virus production was detected as described in the legend to Fig 3. The error bars represent SEM derived from at least two separate experiments performed in triplicate. Mann-Whitney test was used to determine significance difference between the infection with and without FI; * = *P* < 0.05, (n.d.) denotes ‘‘not detectable”.

It is known that the structure of a virion influences the ability of an antibody to neutralize or enhance infectivity [52–55]. Yet, the structure of DENV particles during acute infection is unknown. We therefore attempted to dissect how many prM-containing particles circulate in the blood of DENV-infected humans. To this end, the acute serum samples were added to anti-prM Ab-coated ELISA plates and the extent of virus binding was quantified by qPCR. Unfortunately, despite the high viremia titers in acute sera, the numbers of ELISA captured virions were insufficient to reach the threshold necessary for reliable qPCR measurements. Therefore we used an alternative approach and instead of evaluating the number of prM-containing particles, we now questioned to what extent (partially) immature particles contribute to the ADE profile measured with std DENV preparations. For this approach we take advantage of the fact that antibody-opsonized immature viruses require enzymatic activity of furin in host cells to become infectious [31–33]. The ADE properties were therefore measured in P388D1 cells, treated with decRRVKR-CMK, furin specific. We have used this approach before with monoclonal Abs and found up to 10-fold reduction in viral infectivity following infection of FI-treated cells with Ab-std DENV2 complexes [32, 34]. Prior to the experiment, we first confirmed that the infectivity of immature particles opsonized with polyclonal sera is dependent on the activity of cellular furin during viral entry (Fig 4A). The half-life of FI is approximately 4–8 hours in aqueous solution and therefore it is expected that the compound will not interfere with the maturation of newly assembled virions within infected cells. Indeed, under the conditions used, FI did not affect maturation of newly assembled virions, confirming the short lived nature of the inhibitor (control bars in Fig 4A and 4B). Importantly, inhibition of furin activity in target cells abolished infectivity of immature virions opsonized with immune sera, substantiating that maturation upon entry is a prerequisite for rendering prM DENV-immune complexes infectious. Next we, the ADE properties of std DENV2 opsonized with pooled sera from DF, DHF and DSS patients was tested at a 6400x sera dilution, the dilution that yielded the highest power of enhancement in non-treated cells. Fig 4B shows a significant reduction in viral infectivity in cells treated with FI, indicating that ADE is also caused by immature particles present within the standard virus preparations. Contrary to our hypothesis however, the relative contribution of immature virions and antibodies enhancing their infection did not vary between the different disease severity groups.

## Discussion

In this study we investigated the potential involvement of immature particles in dengue pathogenesis. We tested the ability of acute DENV2 sera obtained from DF, DHF and DSS patients to bind and enhance infectivity of immature DENV2. We found that an average of 20–30% of Abs is directed against the prM protein and bind avidly to immature virions, however no significant difference was observed between the distinct severity groups. Furthermore, similar ADE profiles were obtained following infection with immature virions pre-opsonized with sera from DF/DHF/DSS patients, suggesting that the presence of immature particles and the Abs recognizing them cannot be seen as a predisposing factor for severe disease. It is important to mention that acute sera contain virus particles and therefore, Abs in complex with these particles might have gone unnoticed in our assays. Lastly, our data demonstrate that prM-containing virions within std DENV2 preparations significantly contribute to ADE. Collectively, the results presented within this study indicate that immature particles are not a discriminating factor for disease severity but act as a co-factor in disease pathogenesis.

Previous studies showed that prM Abs are more prone to initiate ADE than neutralization of infection [23, 31, 56]. Additionally, Lai and his colleagues have found that the rates of prM-specific antibody responses are significantly higher in patients with secondary infection than in those with primary infection [21]. These observations led us to hypothesize that prM Abs may exacerbate disease during secondary infection. Despite the semi-quantitative nature of the WB analysis, similarly to other studies [22, 23, 29], we found that a significant fraction of human dengue Abs is directed against the prM protein. Nonetheless, contrary to what we hypothesized, the proportion of prM Abs was not different between DF, DHF or DSS patients. This indicates that despite high levels of prM responses in the secondary dengue infections, there is no direct correlation between the level of prM Abs early in the infection and subsequent disease presentation.

Not only prM but also E-specific Abs can bind and enhance the infectivity of immature virions [32, 33]. To study the overall binding capacity of Abs to immature particles an ELISA was performed. We demonstrated that although Abs present in the acute phase of secondary DENV2 infection were able to bind to immature particles, their titer was not discriminative between the disease presentations. Interestingly however, sera of all severity groups had higher IgG binding titers to immature DENV2 than to std DENV2. Previous studies showed that the human antibody response is dominated by anti-E fusion loop (FL) Abs, which have been described to preferentially bind to immature virions [21, 24, 53]. The higher IgG binding titers to immature particles may therefore reflect the presence of FL Abs in our patient sera. Furthermore, even though we could not asses this directly, the higher IgG titer may hint towards a relatively high number of immature DENV particles in DENV-infected patients. Alternatively, the prM response could be triggered due to the release of cleaved prM products from dengue-infected cells. Another implication of these results is that ELISA–based assessment of dengue-specific Abs with use of std virus preparations underestimates the actual IgG titer.

The capacity of the majority of human DENV-specific Abs to neutralize or enhance sequential DENV infection can be influenced by the extent of the virion’s maturation [54]. *In vitro*, std DENV preparations comprise of a mixture of mature, partially and fully immature particles with the latter two being most prevalent when cultured in C6/36 mosquito cells [18–20]. We here observed that the ADE profile was quite similar for all severity groups regardless whether we used a std or fully immature virus preparations. This implies that immature particles present within std DENV preparations are not a discriminating factor for severe disease. Yet, we do show that immature virions present within std DENV preparations significantly contribute to the overall observed ADE. These results strongly suggest that immature particles that do depend on endosomal furin cleavage for infectivity do contribute to the total viral load observed during secondary dengue infection.

It would have been ideal to include the evaluation of the pre-illness sera samples as neutralizing antibody status just before virus exposure is likely the most relevant for protection from infection [57]. Also it would avoid the confounding effect of the immune-complexes in our assays. Unfortunately, thus far such samples are not available to us in sufficient numbers.

The unforeseen and disappointing results of the Sanofi trail showing that high neutralizing Abs titers are not indicative of DENV2 protection [58] clearly demonstrate that we do not yet fully understand the correlates of protection against DENV. What then determines the development of severe disease? Presently, we also cannot answer this question and based on this and many other studies, it is clear that dengue virus pathogenesis is extremely complex and involves multiple virus and host factors [5, 59, 60]. The infection history of the patient in combination with the current infecting DENV serotype and its maturation state will determine the balance between neutralizing and enhancing Abs. These together with yet unspecified host factors will likely predispose to more severe disease. Evidently, it remains an enormous challenge to fully understand why some individuals are protected against severe disease whereas others do not.

## Supporting Information

S1 Fig Western blot analysis of serum IgG binding to purified immature (prM) DENV2. 1x10^9^ GCPs of purified prM DENV2 was loaded on 12.5% SDS polyacryramide gels under non-reducing conditions. The blot was incubated with 3 serial dilutions of 30 individual sera or with 3 dilutions of pooled sera from 10 DF, 10 DHF or 10 DSS patients. (A) Representative WBs of individual serum samples from 3 disease severity groups (B) Representative WBs of one dilution of pooled serum samples from at least 3 experiments. A human monoclonal prM antibody (hmAb) 75.9 (a kind gift from A. Lanzavecchia. Institute for Research in Biomedicine, Bellinzona, Switzerland) was used as a positive control for prM (and E) band recognition. M denotes marker ladder, The viral proteins are indicated. Note that multiple prM bands that are detected by 75.9 mAb and immune sera have been described before [20, 21] and are thought to represent different glycosylation pattern of this protein.
(TIF)

S2 Fig Concentration of patients sera IgG. Detection of human IgG using sandwich ELISA (Sigma-Aldrich). Each dot represents mean IgG concentration of 1 donor from 3. No statistical differences found between the 3 disease severity groups (Kruskal-Wallis statistic)
(TIF)

S3 Fig Detection of DENV2-specific IgM in the pooled serum samples. Binding of serum IgM to (A) standard (std) DENV2 and (B) immature (prM) DENV2 was analyzed by means of indirect ELISA. For DF, DHF, DSS, 10 individual serum samples were used to create a pool. A pool of 3 primary DENV2 cases were used as a positive control in the assay.
(TIF)

S4 Fig Individual analysis of neutralizing and enhancing capacity of immune sera towards immature DENV2. P388D1 cells were infected with immature DENV2 at MOG 500 in the absence or presence of serially diluted individual immune serum. Panel (A) represent data for DF sera; panel (B) for DHF sera and panel (C) for DSS sera. Virus production was detected as described in the legend to Fig 3. No statistical differences in PFU titers between each dilution of the three groups (One way Anova).
(TIF)
