# Supplementary figures and images for: Antibodies against Immature Virions Are Not a Discriminating Factor for Dengue Disease Severity
Source: PLoS Negl Trop Dis. 2015 Mar 11;9(3):e0003564. doi: 10.1371/journal.pntd.0003564 (PMC4356584; doi:10.1371/journal.pntd.0003564)

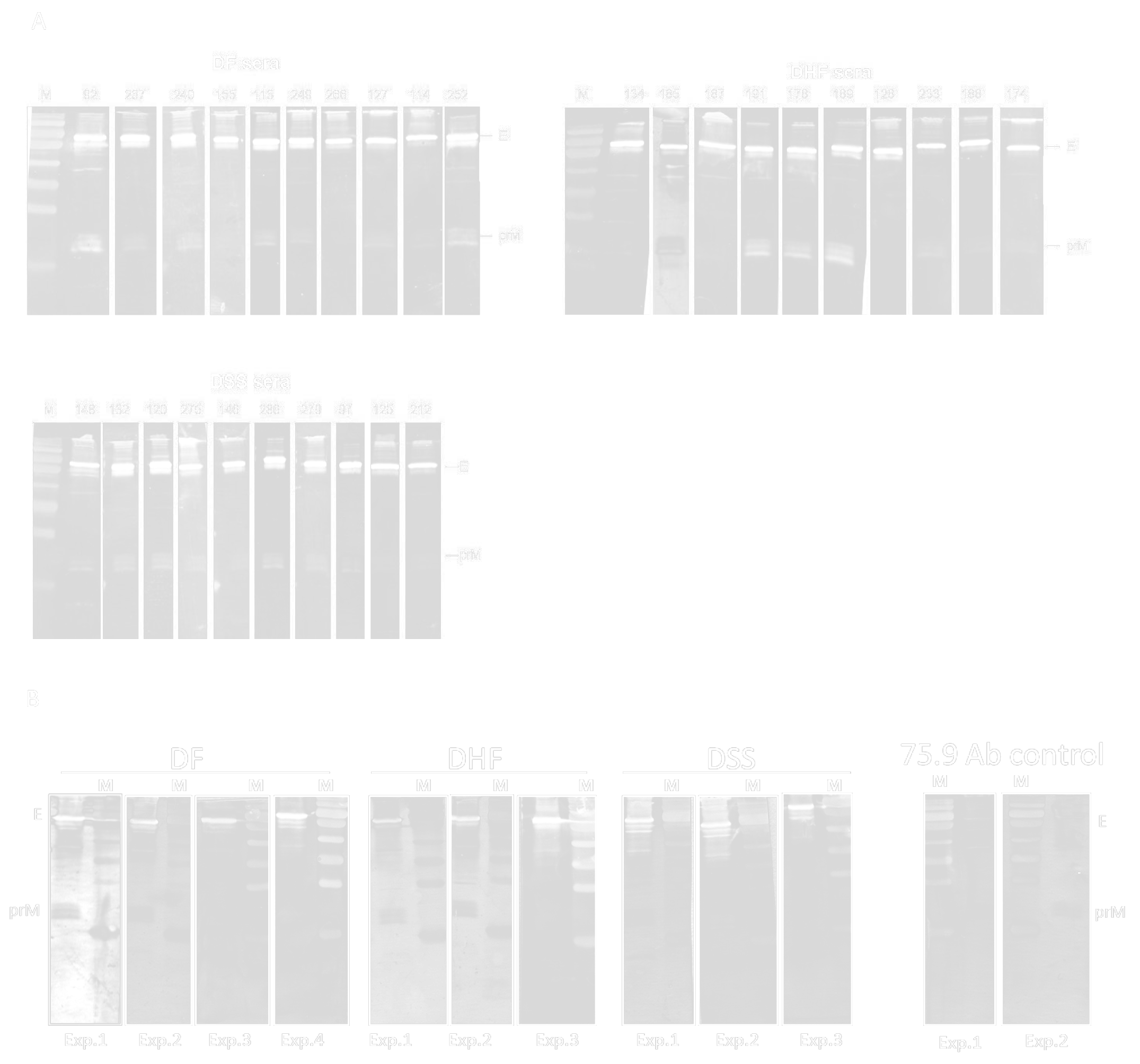

Supplement: S1 Fig — 1x10^9^ GCPs of purified prM DENV2 was loaded on 12.5% SDS polyacryramide gels under non-reducing conditions. The blot was incubated with 3 serial dilutions of 30 individual sera or with 3 dilutions of pooled sera from 10 DF, 10 DHF or 10 DSS patients. (A) Representative WBs of individual serum samples from 3 disease severity groups (B) Representative WBs of one dilution of pooled serum samples from at least 3 experiments. A human monoclonal prM antibody (hmAb) 75.9 (a kind gift from A. Lanzavecchia. Institute for Research in Biomedicine, Bellinzona, Switzerland) was used as a positive control for prM (and E) band recognition. M denotes marker ladder, The viral proteins are indicated. Note that multiple prM bands that are detected by 75.9 mAb and immune sera have been described before [20, 21] and are thought to represent different glycosylation pattern of this protein. (TIF) [file pntd.0003564.s001.tif]

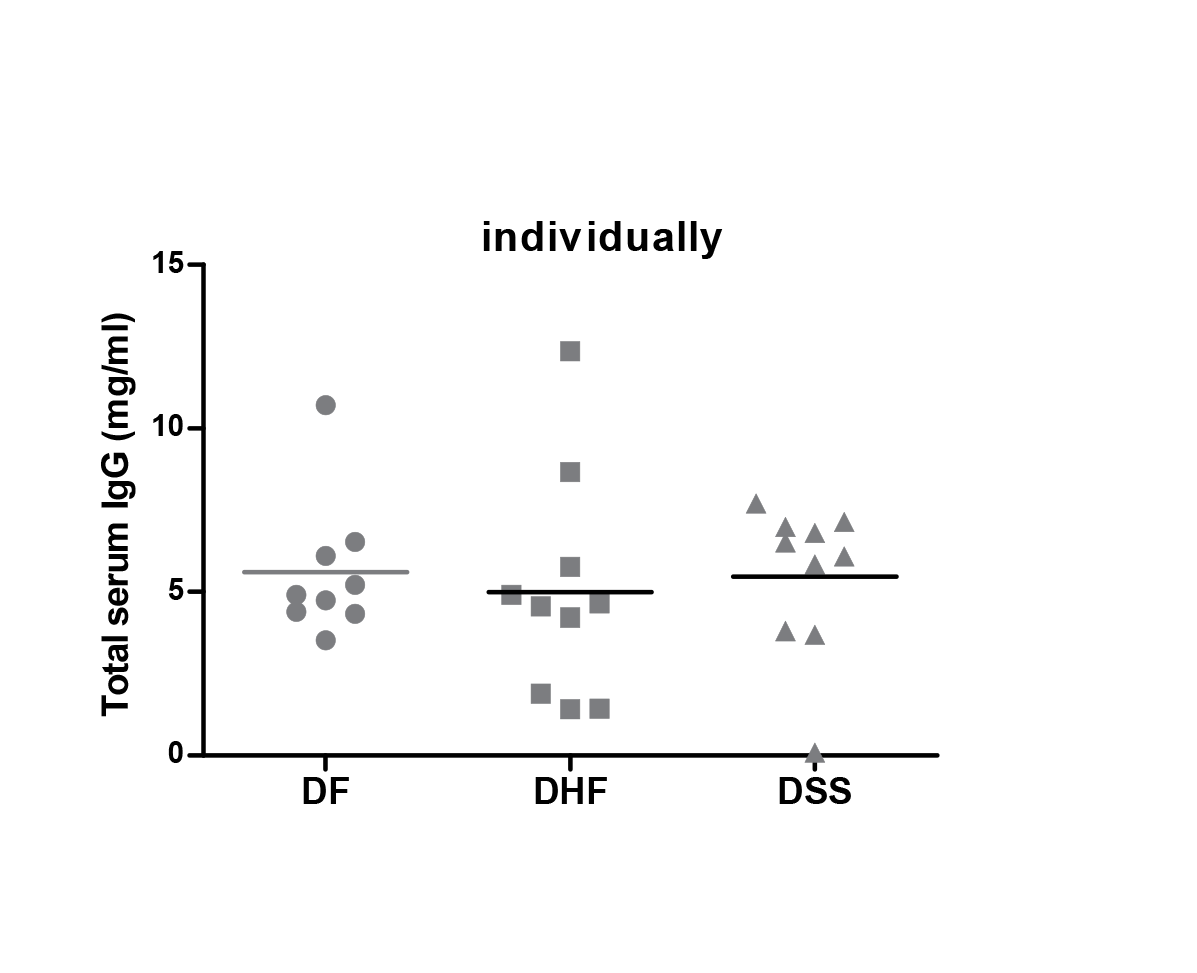

Supplement: S2 Fig — Detection of human IgG using sandwich ELISA (Sigma-Aldrich). Each dot represents mean IgG concentration of 1 donor from 3. No statistical differences found between the 3 disease severity groups (Kruskal-Wallis statistic) (TIF) [file pntd.0003564.s002.tif]

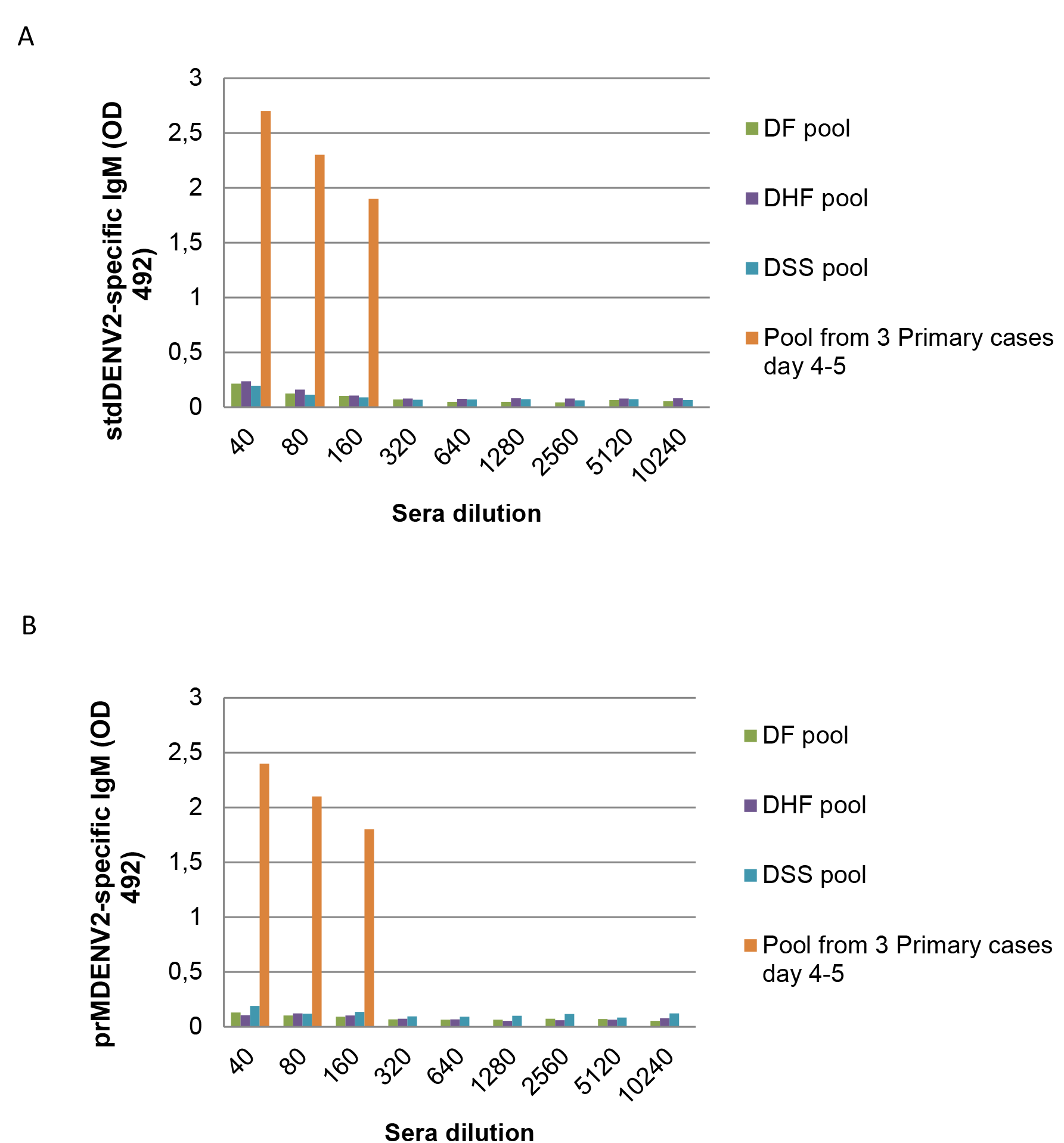

Supplement: S3 Fig — Binding of serum IgM to (A) standard (std) DENV2 and (B) immature (prM) DENV2 was analyzed by means of indirect ELISA. For DF, DHF, DSS, 10 individual serum samples were used to create a pool. A pool of 3 primary DENV2 cases were used as a positive control in the assay. (TIF) [file pntd.0003564.s003.tif]

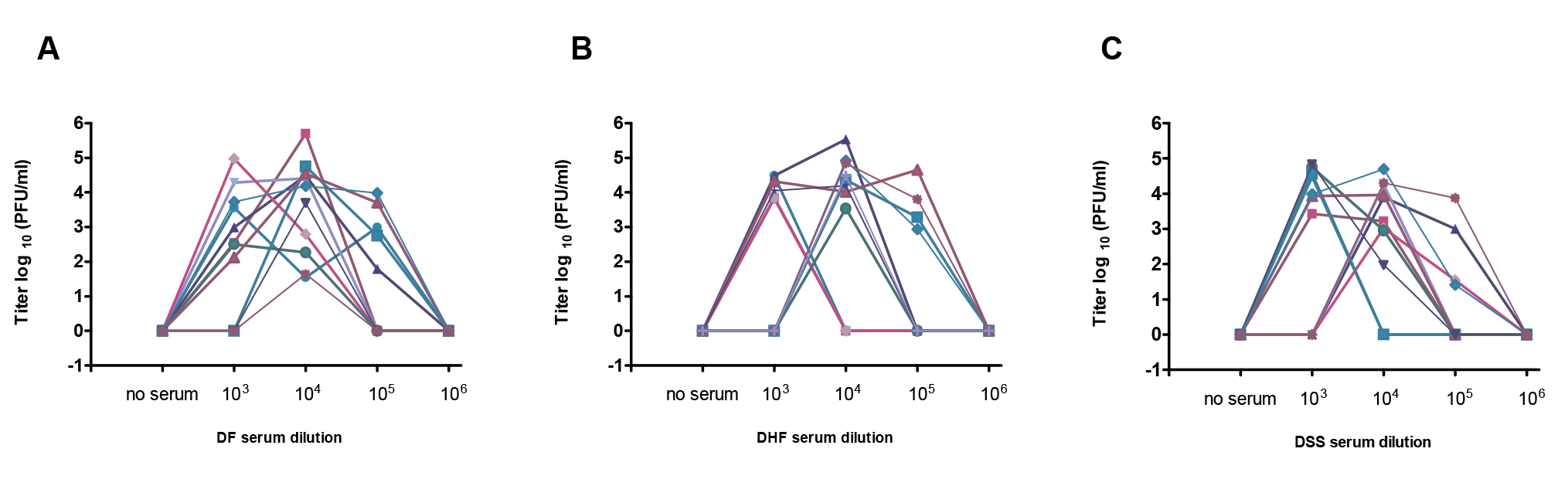

Supplement: S4 Fig — P388D1 cells were infected with immature DENV2 at MOG 500 in the absence or presence of serially diluted individual immune serum. Panel (A) represent data for DF sera; panel (B) for DHF sera and panel (C) for DSS sera. Virus production was detected as described in the legend to Fig 3. No statistical differences in PFU titers between each dilution of the three groups (One way Anova). (TIF) [file pntd.0003564.s004.tif]
